# Supplementary material for: Inverted perovskite solar modules with 99.3% geometrical fill factor via nanosecond single laser patterning
Source: Commun Eng. 2025 Nov 21;4:198. doi: 10.1038/s44172-025-00512-4 (PMC12638776; doi:10.1038/s44172-025-00512-4)
Supplement: Supplementary file 2 — Supplementary Information [file 44172_2025_512_MOESM2_ESM.pdf]

Supplementary information:

Inverted perovskite solar modules with 99.3% geometrical fill factor via nanosecond single laser patterning.

Andrés E. R. Soto[1,2], Vera C. M. Duarte[1,2], Adélio Mendes[1,2], and Luísa Andrade[1,2]*

[1]LEPABE─Laboratory for Process Engineering, Environment, Biotechnology and Energy, Faculty of Engineering, University of Porto, Rua Dr. Roberto Frias, 4200-465 Porto, Portugal.
[2]ALiCE─Associate Laboratory in Chemical Engineering, Faculty of Engineering, University of Porto, Rua Dr. Roberto Frias, 4200-465 Porto, Portugal.

* landrade@fe.up.pt

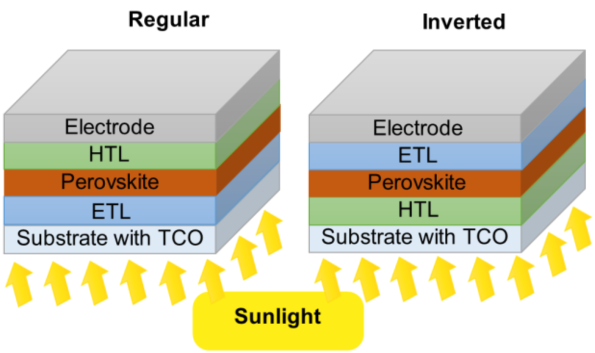

Supplementary Figure 1: Perovskite solar cell architectures.

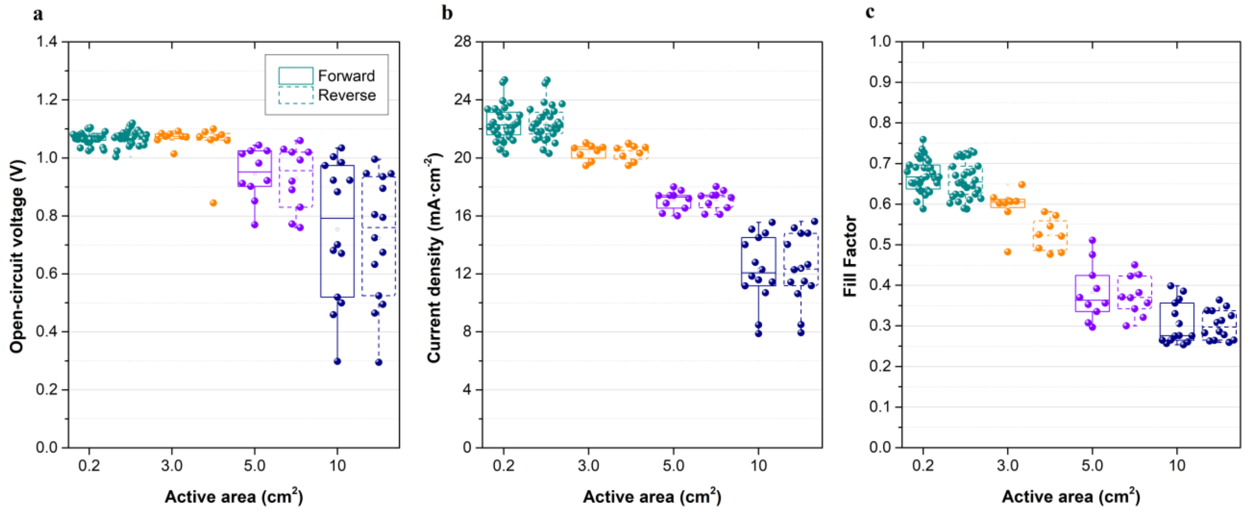

Supplementary Figure 2:Box plot of inverted PSC photovoltaic parameters: a) open-circuit voltage; b) Current density; and c) Fill factor.

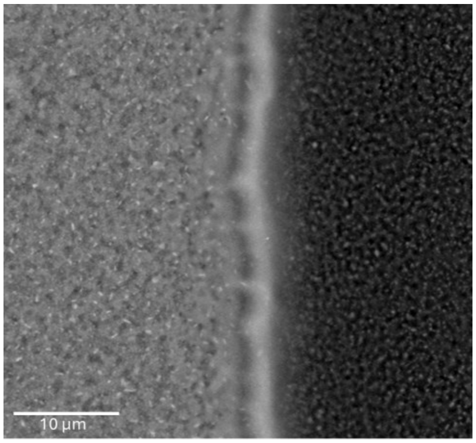

Supplementary Figure 3: SEM image of the P2 scribe border, showing the damaged material caused by the laser pass.

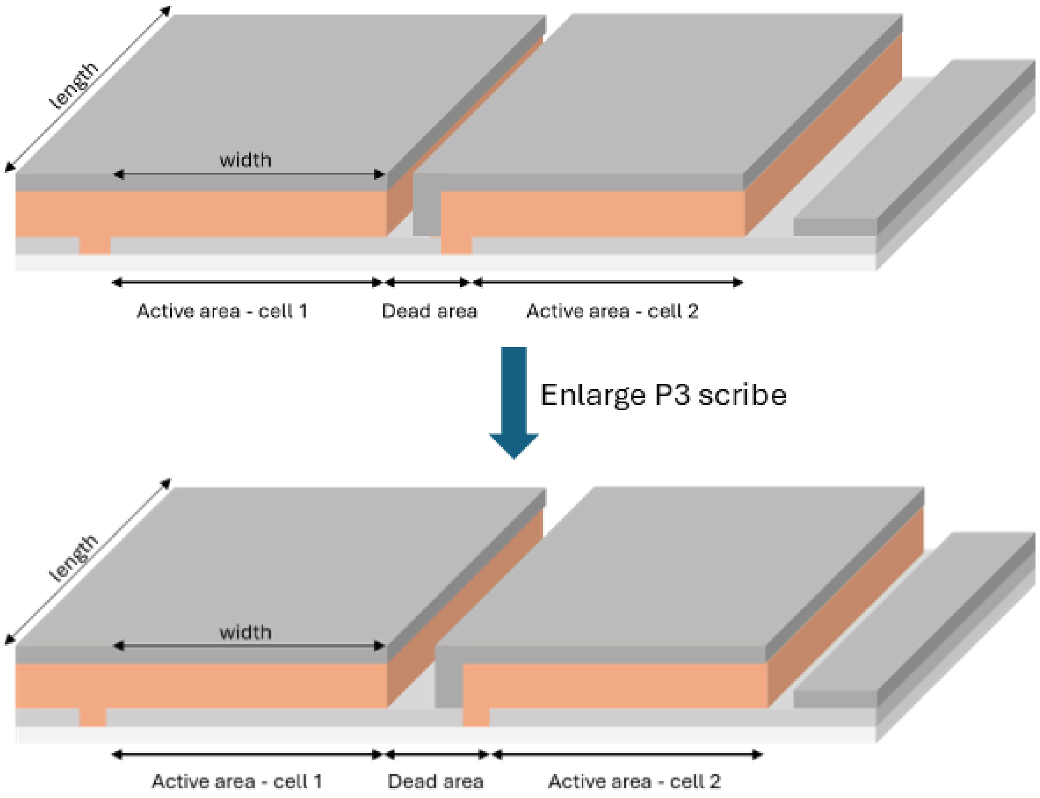

Supplementary Figure 4: Layout of a 2 subcell PSC module showing the enlargement of the P3 scribe (the same strategy was applied to P2 scribe).

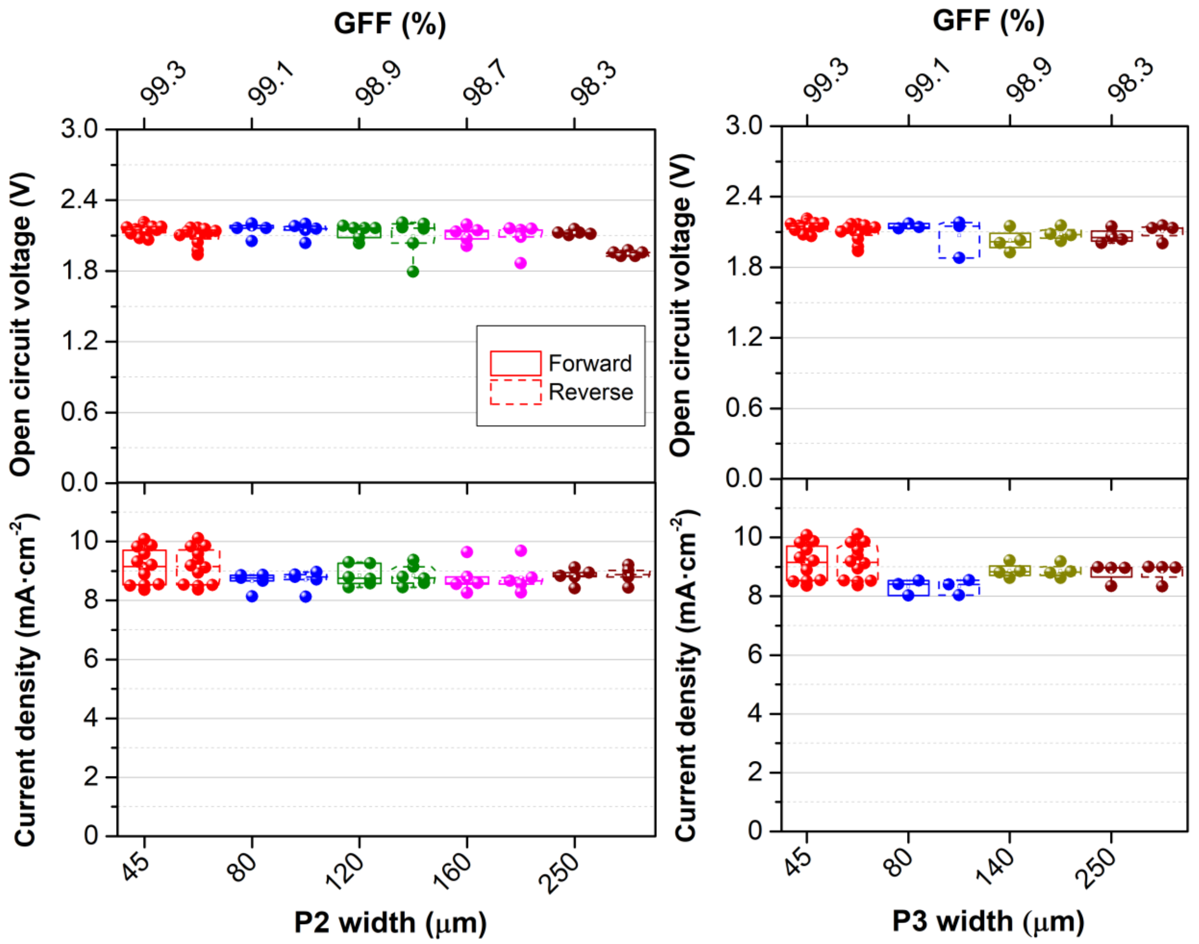

Supplementary Figure 5: Box plot matrix of Voc, and Jsc versus the P2 and P3 width, for 4 cm² inverted PSC modules, and corresponding GFFs. The values represent data from 6 independent batches.

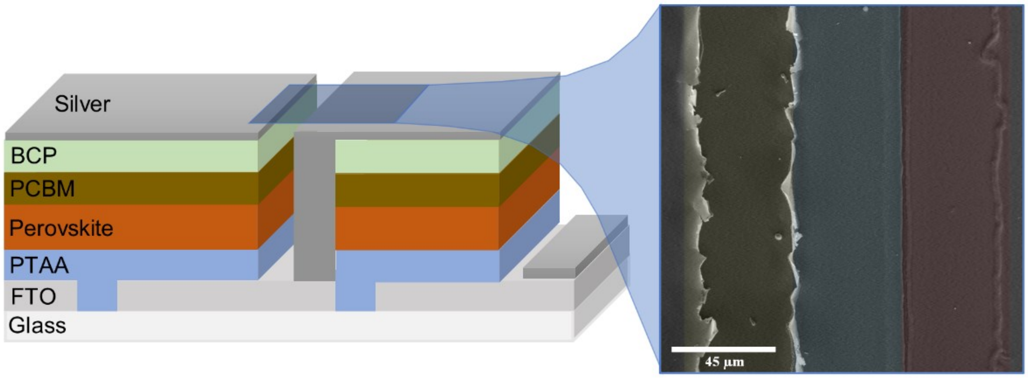

Supplementary Figure 6. Schematic representation of inverted perovskite solar module and SEM image of the interconnection area, where red is P1, blue is P2, and grey is P3.

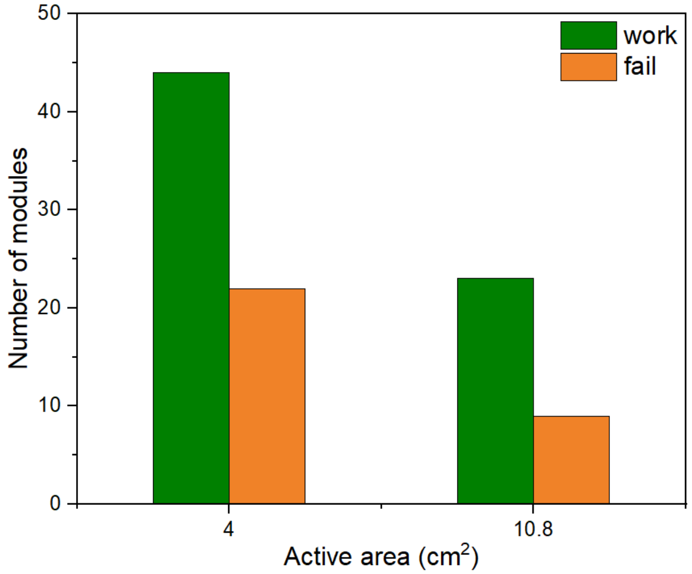

Supplementary Figure 7: Fabrication success rates for prepared PSC modules.
