# Supplementary material for: Inverted perovskite solar modules with 99.3% geometrical fill factor via nanosecond single laser patterning
Source: Commun Eng. 2025 Nov 21;4:198. doi: 10.1038/s44172-025-00512-4 (PMC12638776; doi:10.1038/s44172-025-00512-4)
Supplement: Supplementary file 3 — Photovoltaic Reporting [file 44172_2025_512_MOESM3_ESM.pdf]

nature portfolio

Corresponding author(s): Prof. Luísa Andrade

Solar Cells Reporting Summary

Nature Portfolio wishes to improve the reproducibility of the work that we publish. This form is intended for publication with all accepted papers reporting the characterization of photovoltaic devices and provides structure for consistency and transparency in reporting. Some list items might not apply to an individual manuscript, but all fields must be completed for clarity.

For further information on Nature Research policies, including our data availability policy, see Authors & Referees.

Experimental design

Please check the following details are reported in the manuscript, and provide a brief description or explanation where applicable.

1. Dimensions

Area of the tested solar cells

- [x] Yes — An area of 0.2, 3, 5, and 10 cm2 for individual cells and 4 and 10.8 cm2 for modules.
- [ ] No — *Explain why this information is not reported/not relevant.*

Method used to determine the device area

- [x] Yes — The device area was determined by measuring length and width of individual cell or module with a caliper.
- [ ] No — *Explain why this information is not reported/not relevant.*

2. Current-voltage characterization

Current density-voltage (J-V) plots in both forward and backward direction

- [x] Yes — Provided in Figures 3a and 10b.
- [ ] No

Voltage scan conditions

- [x] Yes — A scan rate of 20 mV·s-1 and a voltage step of 10 mV were used for individual cells, and a scan rate of 50 and 100 mV·s-1 and a voltage step of 10 mV were used for modules.
- [ ] No — *Explain why this information is not reported/not relevant.*

Test environment

- [x] Yes — J-V measurements of non-encapsulated solar cells and modules were performed at ambient conditions. See Characterizations in Materials and Methods section.
- [ ] No — *Explain why this information is not reported/not relevant.*

Protocol for preconditioning of the device before its characterization

- [ ] Yes — *Provide a description of the protocol.*
- [x] No — No preconditioning protocol was used in this work.

Stability of the J-V characteristic

- [x] Yes — Provided in Figure 10d.
- [ ] No — *Explain why this information is not reported/not relevant.*

3. Hysteresis or any other unusual behaviour

Description of the unusual behaviour observed during the characterization

- [x] Yes — Minor hysteresis was observed for the fabricated devices by comparing the forward and backward scanning results. See Figures 3a and 10b.
- [ ] No — *Explain why this information is not reported/not relevant.*

Related experimental data

- [x] Yes — Provided in Figures 3a and 10b.
- [ ] No — *Explain why this information is not reported/not relevant.*

4. Efficiency

External quantum efficiency (EQE) or incident photons to current efficiency (IPCE)

- [x] Yes — Provided in Figure 10c.
- [ ] No — *Explain why this information is not reported/not relevant.*

A comparison between the integrated response under the standard reference spectrum and the response measure under the simulator

- [x] Yes — The integrated JSC values from EQE spectra were comparable to the JSC from J-V measurements, as described in the manuscript.
- [ ] No — *Explain why this information is not reported/not relevant.*

April 2023

For tandem solar cells, the bias illumination and bias voltage used for each subcell

☐ Yes — *Provide a description of the measurement conditions.*
☒ No — *Explain why this information is not reported/not relevant.*

5. Calibration

Light source and reference cell or sensor used for the characterization

☒ Yes — The measurements were made under 1 sun irradiation (AM 1.5, 100 mW/cm2) supplied by a solar simulator (150 W Oriel class A). The simulator was calibrated using a single crystal Si photodiode (Newport, USA).
☐ No — *Explain why this information is not reported/not relevant.*

Confirmation that the reference cell was calibrated and certified

☐ Yes — *Identify the independent certification laboratory.*
☒ No — No certification was performed.

Calculation of spectral mismatch between the reference cell and the devices under test

☐ Yes — *Provide a value of the spectral mismatch and/or a description of how it has been taken into account in the measurements.*
☒ No — *Explain why this information is not reported/not relevant.*

6. Mask/aperture

Size of the mask/aperture used during testing

☒ Yes — A black mask was used in individual cells to define a 0.2 cm2 active area. For the other devices no mask was needed because the active area was defined by laser scribing.
☐ No — *Explain why this information is not reported/not relevant.*

Variation of the measured short-circuit current density with the mask/aperture area

☐ Yes — *Report the difference in the short-circuit current density values measured with the mask and aperture area.*
☒ No — No variation was observed for the devices with the active area defined by laser scribing.

7. Performance certification

Identity of the independent certification laboratory that confirmed the photovoltaic performance

☐ Yes — *Identify the independent certification laboratory.*
☒ No — No performance certification of devices were performed.

A copy of any certificate(s)

☐ Yes — *Certificate copies should be provided in the Supplementary information. Please state the supplementary item number.*
☒ No — *Explain why this information is not reported/not relevant.*

8. Statistics

Number of solar cells tested

☒ Yes — For individual cells, more than 10 batches of 12 cells were performed. For the modules, data refers to 6 batches of 12 modules for 4 cm2 modules and 4 batches of 8 modules for 10.8 cm2.
☐ No — *Explain why this information is not reported/not relevant.*

Statistical analysis of the device performance

☒ Yes — Provided in Figures 3, 7, 8, 10 , S1 and S4.
☐ No — *Explain why this information is not reported/not relevant.*

9. Long-term stability analysis

Type of analysis, bias conditions and environmental conditions

☒ Yes — Long-term stability tests of the fabricated modules was performed storing devices in the dark under an inert atmosphere for over a year.
☐ No — *Explain why this information is not reported/not relevant.*
